# A New Horizon in Hip Health: Using an Innovative Variable Loop Curette for Core Decompression and Bone Marrow Aspirate Concentrate in the Management of Pre-collapse Avascular Necrosis of the Hip

**DOI:** 10.7759/cureus.54763

**Published:** 2024-02-23

**Authors:** Amit Kale, Sushant Kumar

**Affiliations:** 1 Orthopaedics, Dr. D. Y. Patil Medical College, Hospital and Research Centre, Pune, IND; 2 Orthopaedics, Dr. D. Y. Patil Medical College, Hospital And Research Centre, Pune, IND

**Keywords:** total hip arthroplasty (tha), covid-19, avascular osteonecrosis, innovative curette, bmac, core decompression, painful hip, hip joint, avascular necrosis (avn)

## Abstract

Avascular necrosis (AVN) of the femoral head, or osteonecrosis (ON), is a debilitating condition characterized by disrupted blood supply to the hip joint, leading to subchondral bone necrosis, joint collapse, and arthritis. Emerging evidence suggests that the long-term use of corticosteroids, particularly in the context of COVID-19 treatment, may contribute to AVN development.

This case report presents a male in his 50s with bilateral hip pain and a history of corticosteroid use. The patient underwent core decompression (CD) with a bone marrow aspirate concentrate (BMAC) infusion using the innovative curette technique.

Postoperatively, he followed a structured rehabilitation protocol and experienced significant pain relief and improved function. Reviewing existing literature, CD with BMAC using innovative curettes emerges as a promising approach for pre-collapse AVN management, preserving hip function, and delaying the necessity for total hip arthroplasty (THA). This case highlights the potential benefits of this technique in early-stage AVN, emphasizing its role in improving functional outcomes and limiting disease progression.

## Introduction

Osteonecrosis (ON) of the femoral head develops when the femoral head's blood supply is interrupted. It is a typical multifactorial hip disease that causes the subchondral bone to become ischemic necrotic and collapse, as well as progress to more progressive stages of joint space constriction and arthritis, all of which have an impact on quality of life and functional status [1].

The new coronavirus has spread and changed into several forms since it originally emerged, having a huge influence on people's lives. One of the side effects is avascular necrosis (AVN), which, if addressed, may result in disastrous situations and bone collapse. Many lives have been spared due to the use of corticosteroids in the short-term treatment of moderate-to-severe SARS-CoV-2 infections. Corticosteroid usage over an extended period of time is linked to a variety of adverse effects. A common adverse effect of steroids is AVN of the femoral head, which is made worse by the illness itself.

In terms of demographics, the illness is particularly concerning as it often impacts individuals who are young or middle-aged and have a substantial remaining life expectancy [2]. To safeguard the femoral head and delay arthroplasty as much as possible, it is vital to intervene in the early stages of the disease's development. According to Kamani et al. found that the effects of COVID-19 on steroidal injections led to the development of bilateral AVN of the femoral head in a young male [3]. This condition impeded his daily activities and posed challenges in managing the undesirable side effects of steroidal injections [4].

Various joint-preserving treatments, such as bone marrow aspirate concentrate (BMAC) injection, core decompression (CD), muscle pedicle grafting, bone grafting, recombinant bone morphogenic proteins, and platelet-rich plasma, are currently being used for management. Total hip arthroplasty (THA) is now the gold standard treatment for advanced AVN patients. Despite the outstanding outcomes of arthroplasty, the prosthesis' longevity is still a problem in the young as well as the middle-aged population, necessitating revision surgery in the majority of instances [5]. As a result, different methods have been used to postpone the development of arthritis and any following THAs, including rotational osteotomies, vascularized bone grafting, and CD with BMAC.

Increased osteoprogenitor cell presence in the necrotic region as a result of BMAC injection will help with tissue repair [6-7]. According to Ficat-Arlet staging, CD alone has been shown to be effective in about 84 to 95% of Stage 1 individuals and 65% of Stage 2 individuals; results are uncertain in later stages [8]. BMAC has now been utilized as an accessory and has produced more favorable outcomes in AVN’s early stages. Since the subchondral bone had already fallen beyond the crescent stage, investigations of BMAC were able to evaluate the operation's potential as a hip-preserving surgery in its later stages.

## Case presentation

In our case study, a male in his 50s complained that he had been experiencing discomfort in both hip joints for one and a half years. He was apparently well one and a half years ago when he started experiencing pain in the bilateral hip joints (right > left), which was insidious in onset, moderate in severity, deep aching in character, continuous in nature, aggravated on walking, climbing stairs, and squatting, and relieved on rest and pain medication. His past history was significant in terms of steroid intake and alcohol consumption.

**Figure 1:**
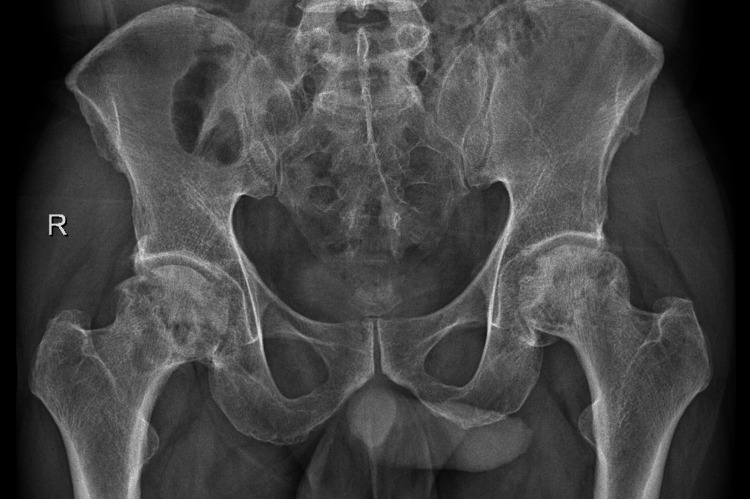
A plain radiograph of the pelvis and bilateral hip joints was obtained, which depicted mild sclerosis, cystic changes, and changes in osteonecrosis

**Figure 2:**
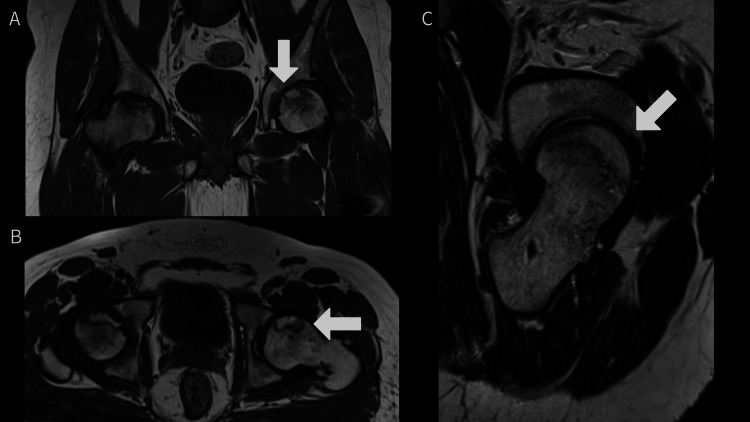
An MRI of the pelvis and bilateral hip joints was obtained, which showed features of Grade 2 AVN on the left side (marked with an arrow) and Grade 3 AVN on the right side MRI: magnetic resonance imaging; AVN: avascular necrosis

We obtained a plain radiograph of the pelvis and bilateral hip joints, which depicted mild sclerosis and cystic changes on the left side (Figure 1). An MRI of the pelvis and bilateral hip joints was obtained, which showed features of Grade 2 AVN on the left side and Grade 3 AVN on the right side (Figure 2A-2C).

The patient underwent a CD procedure with BMAC for the left side of the hip joint, employing an innovative curette. This was preceded by thorough preoperative investigations that revealed no significant abnormalities. Additionally, a pre-anesthesia checkup was conducted, and the procedure was carried out with the patient's informed consent. After three months, the patient underwent THA on the other side, as it was Grade 3 AVN on the other side.

Procedure

**Figure 3:**
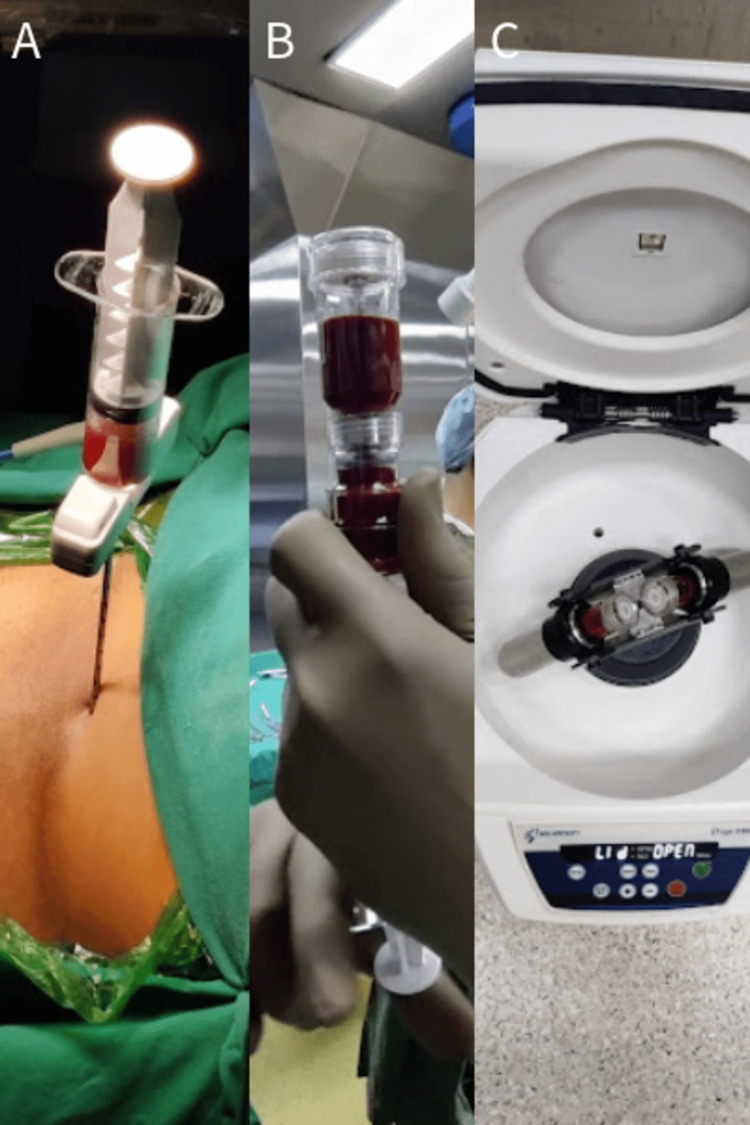
(A-B) Aspiration of bone marrow concentrate from the iliac crest. (C) Centrifugation of bone marrow concentrate

**Figure 4:**
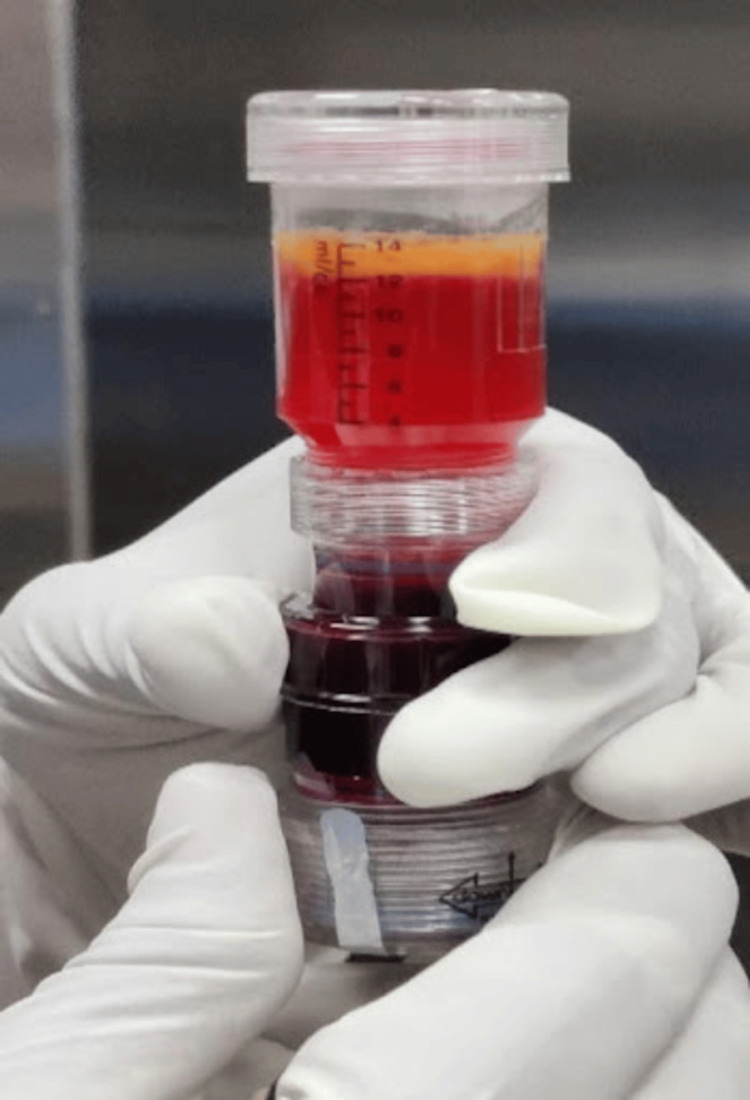
The separation of stem cell-rich components after centrifugation

The patient was taken in a supine position on the fracture table, with scrubbing, painting, and draping done. Bone marrow aspiration was done from the iliac crest, and 60 ml of blood was harvested in a pre-lock syringe patented by the company Tricel that was pre-treated with anticoagulant (Figure 3A-3B). To separate the different components, centrifugation was used on the aspirate (Figure 3C). Stem cell-rich components after centrifugation were obtained (Figure 4).

**Figure 5:**
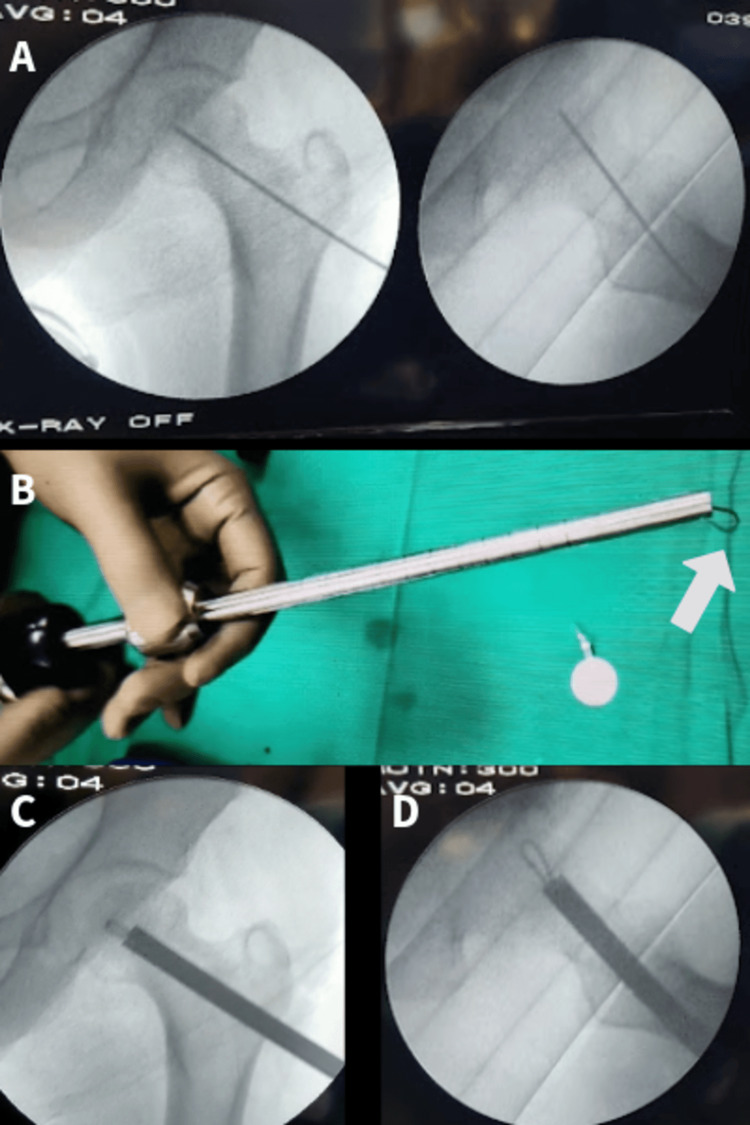
(A) Path of CD. (B) Curette with a loop blade (marked with an arrow). (C-D) CD by a loop blade in anteroposterior and lateral views, respectively CD: core decompression

A 5 cm lateral incision taken over the lateral aspect of the proximal femur, 5 cm distal to the greater trochanter, was taken in a supine position under careful aseptic precautions on the fracture table on the left side. The vastus lateralis muscle was split, and a path of CD was made (Figure 5A). A 6 mm core drill was used to remove the cortical window over the lateral femur. A specialized drill that had a variable curette loop (Figure 5B) and could be adjusted from outside was introduced under C-arm guidance in the superior-anterior necrotic area of the femoral head (Figure 5C-5D). It was then progressively opened from 4 mm to 12 mm loops to achieve complete removal of necrotic bone from the head. This is very innovative to be able to remove larger necrotic areas through smaller holes. A thorough wash was given, and all necrotic bony material was removed. The stem cell-rich BMAC was infused with a spinal needle. The incision was stitched up in layers after a bone plug was applied. A bone plug was applied to fill the bony defect in the lateral wall of the femur after performing CD.

Postoperative rehabilitation

**Figure 6:**
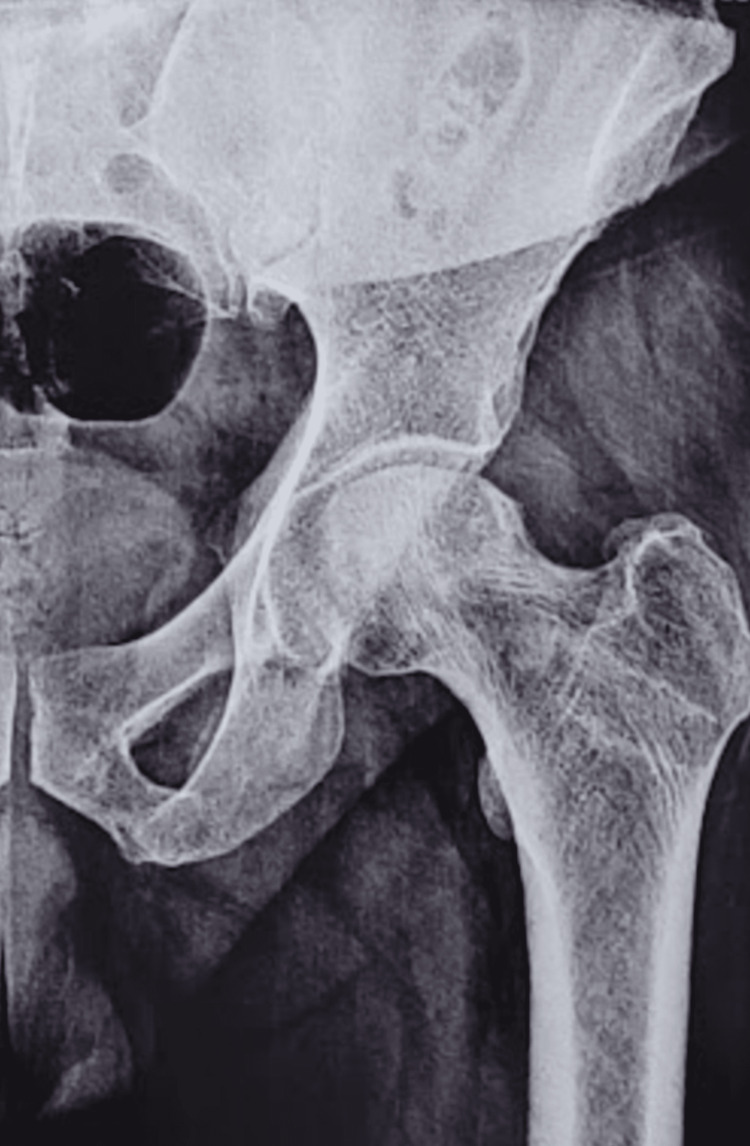
Three-month postoperative anteroposterior view X-ray of the left hip

In our case, a male in his 50s with a history of pain and an inability to walk presented with bilateral AVN of hip joints. The procedure for CD with an infusion of BMAC was uneventful. Preoperatively, the patient's score was evaluated using a VAS score of 8. Postoperatively, the patient was made a partial weight bear after six weeks and a full weight bear after 12 weeks, and the VAS score was 3. After three months of follow-up, a postoperative X-ray was obtained (Figure 6). After three months, the patient underwent THA on the other side, as it was Grade 3 AVN on the other side.

At present, the patient is pain-free, is able to do his day-to-day activities, and walks without a walker or a stick, which is consistent with the studies discussed earlier.

## Discussion

Hip AVN is a degenerative condition that, if left untreated, results in femoral head deformation and arthritis, both of which may be effectively treated with hip arthroplasties. According to theories about its pathogenesis, the femoral head's osteogenesis declines as a result of fewer mesenchymal mononuclear cells, and the injection of these cells may thus aid in trabecular regeneration.

The skeletal foundation of the hip relies on mononuclear stem cells, present in BMAC. These cells, when introduced, have the capacity to replace aging and damaged cells, transforming them into bone-forming cells and thereby maintaining structural stability. A potential therapeutic approach for pre-collapse stages could entail replenishing a substantial quantity of hip stem cells during decompression. This process aims to establish a new reservoir of lineage cells that have the potential to develop into the necessary cells for the hip's normal physiological function [8].

With an average follow-up of 5.8 years, Tomaru et al. assessed 31 AVN patients who had received CD and BMAC treatment. A higher Japanese Orthopaedic Association score and a lower pain score (both P<0.05) indicated that the patients had improved. Three patients (9.6%) required hip arthroplasties, and 11 out of 31 hips with more extensive necrosis collapsed. Previous studies have described the natural course of hips with comparable AVN, but the authors of this study claim to have shown a decreased incidence of collapse in hips with a wider region of necrosis (inhabiting more than the medial 2/3rd of the weight-bearing part). Overall, the research demonstrated that CD with BMAC is a less invasive modality with superior outcomes in the early phases of AVN [9].

At an average follow-up of sixteen months, Talathi et al. used this approach in 43 hips of 28 patients and saw a substantial reduction in VAS score from 7.8 preoperatively to 2.5 after surgery” (P<0.0001). In addition, 40 hips on serial radiographs showed no advancement, with 78% of their patients reporting pain reductions of greater than 50%. At a mean time of seventeen months after surgery, three instances of femoral head collapse required arthroplasty. The authors came to the conclusion that this approach might halt disease development and significantly reduce symptoms [10].

CD of the femoral head has been shown to adequately treat patients with pre-collapse disease without changes on radiographs (Steinberg Stage 1); however, when the areas of ON become visible on radiographs (Steinberg Stage 2), CD alone has been associated with disease progression. In order to improve the rate of hip preservation, Hernigou et al. [11] began combining hip decompression with BMACs, recently reporting a mean 25-year follow-up showing CD combined with BMAC yielded 72% survivorship as compared to decompression alone at 28.32%. In the long-term follow-up study by Hernigou et al. [11], the authors noted that patients with advanced disease (Steinberg Stage 2) were at increased risk of progression to THA.

In 66 hips of 52 patients, Einhorn et al. employed CD with BMAC, and after two years, they found a 63% development in overall Western Ontario and McMaster Universities Osteoarthritis Index scores (down from 36 at baseline to 13 at two years), P=0.001. Joint stiffness and pain symptoms considerably decreased, and the SF-12 and EQ-5D QoL scores also considerably decreased (P<0.001). With 11 hips left unfollowed, the hip survival rate was 75% (41/55), and only 14/55 hips required total hip replacement. The authors observed an overall positive result and deemed CD+BMAC a suitable method for Stage 1 and 2 AVN hips. Nevertheless, although CD alone has been shown to be very effective in Stage 1 instances, BMAC should preferably be utilized in later stages of AVN [12].

## Conclusions

The integration of CD with BMAC using an innovative curette presents a promising therapeutic avenue for addressing AVN of the hip, particularly in the pre-collapse stages. The potential effectiveness of this combined approach is evident in its capacity to enhance functional scores and impede the radiological progression of the disease. The inclusion of postoperative physiotherapy is integral to maximizing the benefits of interventions like CD with BMAC for AVN of the hip. It promotes functional recovery, reduces complications, and empowers patients to actively contribute to their rehabilitation, ultimately enhancing the overall success of the surgical procedure.
